# Virtual Fracture Clinic Patients’ Satisfaction Outcomes

**DOI:** 10.7759/cureus.60528

**Published:** 2024-05-17

**Authors:** Mohamed Nagy, Christos Kitsis, Samar Abdelhameed, Gur Aziz Singh Sidhu, John Atherton, Neil Ashwood

**Affiliations:** 1 Trauma and Orthopaedics, Cairo University Hospitals, Kasr Alainy, Cairo, EGY; 2 Trauma and Orthopaedics, Manchester University Hospitals NHS Foundation Trust, Manchester, GBR; 3 Trauma and Orthopaedics, University Hospitals of Derby and Burton NHS Foundation Trust, Burton, GBR; 4 Radiology, Cairo University Hospitals, Kasr Alainy, Cairo, EGY; 5 Radiology, Manchester University Hospitals NHS Foundation Trust, Manchester, GBR; 6 Trauma and Orthopaedics, University Hospital Lewisham, London, GBR; 7 Research Institute, University of Wolverhampton, Wolverhampton, GBR

**Keywords:** telemedicine, orthopaedics, questionnaire, patient satisfaction, virtual fracture clinic

## Abstract

Background

During the COVID-19 pandemic, there was a need to balance optimum treatment service and the safety of patients and hospital staff. The British Orthopaedic Association recommended a virtual fracture clinic to give the right first-time decision and to minimize patient exposure to disease. This study aimed to evaluate the patients’ satisfaction outcomes for the service provided through the virtual fracture clinic.

Methodology

From January to May 2022, all patients seen by the staff in the Emergency Department (ED) at Queens Burton Hospitals were enrolled in a prospective study. An Excel spreadsheet was provided to both ED personnel and the orthopaedic team for accessibility. Patients were continually added to the spreadsheet, and their cases were reviewed by the on-call consultant to devise treatment plans. A satisfaction questionnaire was collected from patients about their virtual clinic experience as a route to provide treatment service.

Results

The study comprised 150 patients, with an average age of 40 years. Distal radial fractures represented one-third of the cases. Different modalities of management were offered such as a sling, splint, cast, or referral to physiotherapy. Around 75% of cases were satisfied, understood the advice given over the phone, and were not required to attend the actual clinic. The remaining quarter attended the clinic either for further reassessment by an orthopaedic surgeon or for discussion of their injury as they could not get the full message over the phone due to fast calls, hearing struggles, or just listening to messages.

Conclusions

The virtual fracture clinic is an effective standalone service that gained around 75% satisfaction in patients’ outcome questionnaires. It saved the actual fracture clinic slots and hospital resources. It is recommended to be part of the standard daily practice throughout the United Kingdom.

## Introduction

The integration of virtual clinics in orthopaedics marks a fundamental change in how musculoskeletal care is delivered. With advancements in technology, patients are increasingly looking for convenient and effective methods to access healthcare. Virtual clinics offer patients the opportunity to remotely consult with orthopaedic specialists, eliminating the necessity for travel and decreasing waiting periods [1].

The virtual fracture clinic (VFC) was first introduced in Glasgow Royal Infirmary in October 2011 [2]. It was a collaboration between the Department of Orthopaedic Surgery and the Department of Emergency Medicine [3]. This procedure did not demand any additional time and resources from the Emergency Department (ED) personnel. It yielded substantial advantages for the ED by establishing treatment pathways. These pathways effectively curtailed the unnecessary return visits of patients to in-person fracture clinics for the review of stable, self-limiting injuries [3].

Furthermore, virtual orthopaedic clinics have proven effective in improving patient outcomes and decreasing healthcare expenses. Utilizing telemedicine platforms, orthopaedic specialists can remotely evaluate and diagnose various musculoskeletal conditions, ranging from acute injuries to chronic degenerative diseases [4].

Research conducted by Smith et al. (2020) revealed that patients who engaged in virtual orthopaedic consultations experienced notably shorter wait times than those attending traditional in-person appointments, resulting in enhanced patient satisfaction and adherence to treatment protocols [1].

A systematic review conducted by Gilbert et al. (2021) revealed that virtual consultations in orthopaedics yielded similar clinical outcomes to in-person visits, accompanied by reduced rates of hospital admissions and fewer unnecessary imaging studies, thereby resulting in cost savings for healthcare systems [4].

This study aimed to evaluate the efficiency and patient satisfaction of the VFC at Queens Burton Hospital from a patient’s perspective.

## Materials and methods

This study was conducted at Queens Burton Hospital in the United Kingdom to evaluate the orthopaedic VFCs between January 2022 and May 2022, which included three hospitals, i.e., Queens Burton Hospital, Samuel Johnson Community Hospital, and Sir Robert Peel Community Hospital. Due to the COVID-19 pandemic, and according to the British Orthopaedic Association (BOA) guidelines and statement [5,6], the Orthopaedic Department has switched the service from the traditional face-to-face (F2F) fracture clinic to the VFC pathway which was triaging all patients referred from the ED with orthopaedic-related injuries.

The virtual clinic at Queen’s Hospital Burton and Derby Hospitals Trust was created in 2020 to allow our orthopaedic healthcare professionals to review and treat patients virtually during the COVID-19 pandemic, enforce social distancing, and protect vulnerable patients.

Patients were triaged at the VFC into the following three categories: (1) patients who needed further assessment were booked either at a general fracture clinic or a specialized clinic according to the injury type; (2) referral to physiotherapy; or (3) discharge.

Since 2020, many social and travel restrictions have been lifted by the government, allowing patients to have F2F appointments with orthopaedic healthcare professionals for orthopaedic-related conditions. We have continued to run our virtual clinic to see patients who have been transferred from ED to orthopaedics for further investigation and treatment.

A questionnaire was created for that purpose [7,8]. Patients verbally consented to participate in the study by filling out the questionnaire. All gathered data were anonymized and securely stored on a password-protected computer. The questionnaire was divided into the following three sections: questions 1-4 enquired about the injury, questions 5-8 asked about the VFC experience, and, finally, questions 9-13 enquired about the preceding F2F fracture clinic outcome (Appendix 1).

Patients were also encouraged to provide suggestions for enhancing virtual clinic experiences. Additional data, including patient demographics (age, gender), diagnosis, actions taken during the virtual clinic, and recovery and return to work, were retrieved from our local electronic health records system. Patients were excluded from the study if they were unreachable or required language translation services. Data were analysed using Microsoft Excel (Microsoft Corp., Redmond, WA, USA).

## Results

**Figure 1:**
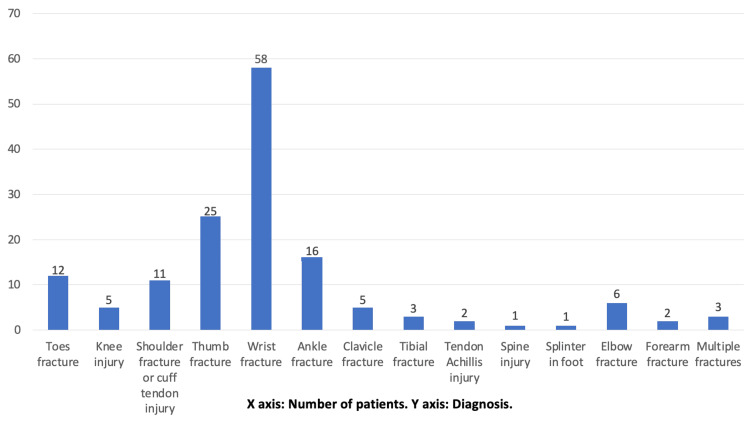
Different injuries presented through the virtual fracture clinic.

The study included 150 patients, who presented between January and May 2022. The average age was 40 years (range = 4 to 81 years). Females represented 55% while males 45%. The patients presented with different diagnoses. As shown in Figure 1, more than one-third of the patients came with a fracture of the wrist.

**Figure 2:**
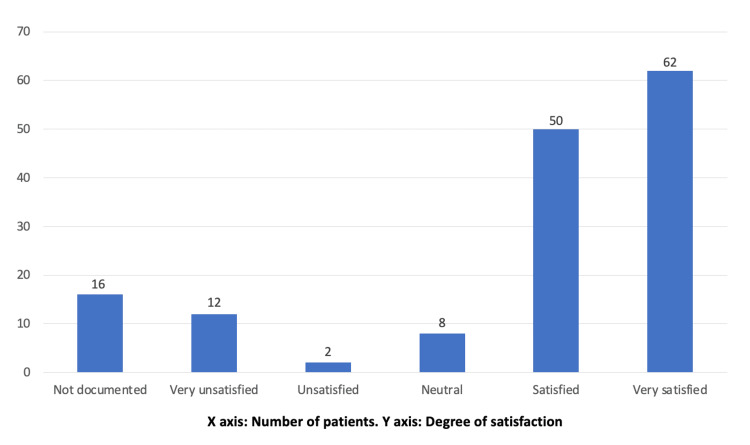
Satisfaction with phone calls at the virtual fracture clinic.

More than 90% of the patients were treated conservatively, with only nine patients requiring day-case admission for surgery. Overall, 67 patients were discharged directly from the VFC by sending the patients letters with their diagnosis and the required splint, sling, or review in the nurse-led clinic. Seven patients were referred directly to physiotherapy or hand therapy to take over care for rehabilitation. Around 44% (67 patients) were booked an F2F appointment in the fracture clinic for further scans and cast application. Nearly most of the patients (88%) understood the management plan over the phone at the VFC. Figure 2 shows the patients’ satisfaction with their VFC experience, with almost 75% of patients reporting being satisfied with the VFC. Unsatisfied patients reported the call being too quick, struggling to hear, or being keen for further F2F assessment as the reasons for their unsatisfaction. By the time of filling out the questionnaire, 80 (53%) patients stated that they had received the VFC letter with the management plan, with 90% being satisfied by the letter’s content.

Regarding the patients who were asked to attend F2F appointments in the fracture clinic, 19 patients out of 67 (28%) reported some difficulties attending in the form of a long way to travel, difficulty in arrangement due to other commitments (e.g., school), or mobility issues related to other injuries. Nearly all patients who attended the fracture clinic understood the management plan and were satisfied with the new pathway of management. The overall satisfaction from the patients’ point of view regarding the whole pathway (VFC in addition to the F2F fracture clinic) was around 90%, with nearly 50% returning to work/normal daily activity by the time of filling out the questionnaire.

The VFC helped to hit the 72-hour target set by the BOA Standards for Trauma guidelines, achieved early therapy intervention, avoided unnecessary attendances, reduced DNAs (Did Not Attend) from 82 to 6, and reduced complaints regarding the fracture clinic appointments from 72 to 22 when comparing this period during 2020 and the similar one in 2019 before the VFC pathway was applied.

Appendix 2 shows the agreed upper limb virtual fracture clinic guidelines applied at Queens Burton Hospital.

## Discussion

The inception of orthopaedic VFCs was initially done for minor orthopaedic injuries such as mallet fractures [9] but now represents a pivotal advancement in fracture management, fundamentally reshaping customary patient care methods. Originating as a solution to address the increasing demand for orthopaedic services and the imperative to enhance efficiency, VFCs swiftly emerged as a practical substitute for traditional, in-person consultations. This transition to virtual care delivery was made feasible by strides in telemedicine technologies, empowering clinicians to remotely evaluate fractures by examining radiographs and clinical data [10].

In this study which included 150 patients, the vast majority of patients, exceeding 90%, received conservative treatment, with only nine individuals requiring day-case admission for surgery. Around 74 patients were discharged directly from the VFC, receiving their diagnosis and necessary equipment, or being referred to physiotherapy. The remaining 67 patients were scheduled for an F2F appointment in the fracture clinic for additional scans and further management. Patients were satisfied with their VFC experience, with nearly 75% expressing satisfaction.

In the study by Jenkins et al. (2016), staffing costs experienced a modest increase of 4% in comparison to the national rise of 16%. Concurrently, the total rate of outpatient department attendance declined by 15% compared to a 5% reduction nationally. The implementation of the VFC system demonstrated a more efficient utilization of staff resources compared to national data, hinting at the potential for substantial cost savings if adopted on a wider scale nationally [10].

The documented potential advantages for patients are increasingly supported by evidence from VFCs and elective follow-up appointments. This evidence indicates enhanced patient satisfaction stemming from decreased travel and waiting times [11].

A comprehensive systematic review done in 2020 including 15 studies involving 11,921 patients showed the effectiveness of VFCs, with approximately two-thirds of the patients being satisfied by their management, thus obviating the need for F2F clinic visits [12].

Additionally, the study revealed that VFCs offered a cost-effective approach for handling stable fractures, yielding satisfactory clinical outcomes and high levels of patient satisfaction. Notably, while existing evidence strongly supports the use of VFCs for certain injuries such as fifth metatarsal fractures and radial head and neck fractures, there remains a dearth of data supporting their efficacy for other specified injuries with simple or stable fracture patterns [12,13].

Furthermore, the virtual clinics moved a step forward to include elective orthopaedic patients during the recovery and rehabilitation phases. A meta-analysis published in 2022, including 11 studies with 1,054 patients, demonstrated that telemedicine is poised to play a crucial role in healthcare delivery both during the COVID-19 pandemic and into the future, aiming to provide safe, efficient, and timely care to orthopaedic patients. The data suggested that virtual clinics proved to be largely equivalent in effectiveness to traditional in-person consultations for elective orthopaedic patients during their recovery and rehabilitation phases. Nonetheless, additional research is warranted to assess its efficacy in the initial assessments [14].

Another observational study was conducted to compare the clinical outcomes of new patients who were electively referred to orthopaedic virtual clinics in January and February 2021 with those attending F2F clinics during the same period in 2020. During the corresponding timeframe, a greater number of patients were reviewed in virtual clinics compared to traditional F2F consultations (821 vs. 499). However, virtual clinics resulted in a significantly higher proportion of patients being scheduled for follow-up appointments (78.3% vs. 37.3%), while fewer patients received outcomes leading to definitive interventions or discharge [15].

The efficacy of virtual clinic appointments for reviewing new patients remains uncertain. Despite the increasing utilization of virtual clinics within the National Health Service, the latest study suggested a potential delay in patients’ clinical progression of the new elective patients, thereby impeding healthcare delivery [15].

## Conclusions

The current study demonstrated the considerable efficacy of the VFC in effectively managing patients referred from the ED, with almost 75% expressing satisfactory outcomes. The incorporation of video call setups could potentially enhance the quality of VFCs and lead to improved patient outcomes. Future research endeavours are encouraged to further assess the utilization of telemedicine in this context.

## Appendices

 Appendix 1

**Table 1:** The questionnaire. QHB = Queens Burton Hospital; Q = Question; ED = Emergency Department; GP = general practitioner

Currently, we are undertaking a study into the efficiency and patient satisfaction of our virtual clinic from a patient’s perspective. Therefore, we are interested in hearing your views about our virtual clinic in this anonymous patient questionnaire. Thank you in advance for providing your views on this patient questionnaire. If you have any further questions about this questionnaire or study, please contact the fracture clinic at QHB.	
Q1	What happened after you recently attended the hospital ED due to your Orthopaedic-related condition? □ Discharged after you received treatment advice from the Orthopaedic surgeon □ Physiotherapy or hand therapy □ Plaster or splint □ Surgery □ Other, please specify: ....................................................................................................................................................
Q2	What is your recent orthopaedic-related condition e.g. broken bone? Which part of your physical body was affected? ........................................................................................................................................................................................................................................................................................................
Q3	Have you recovered from your recent orthopaedic-related condition? □ Yes (please continue to Q4) □ No (please continue to Q5)
Q4	How long did it take for you to recover from your recent orthopaedic-related condition? □ <1 week □ 3–4 weeks □ 1–2 weeks □ 4–6 weeks □ 2–3 weeks □ >6 weeks
Q5	Did you understand the advice given over the telephone by the nurse? □ Yes □ No
Q6	How satisfied were you with your phone call? □ Very unsatisfied □ Unsatisfied □ Neutral □ Satisfied □ Very satisfied Why? ....................................................................................................................................................
Q7	Did you receive a letter about your condition? □ Yes □ No
Q8	How satisfied were you with your letter? □ Very unsatisfied □ Unsatisfied □ Neutral □ Satisfied □ Very satisfied Why? ....................................................................................................................................................
Q9a	Did you experience any difficulties attending your fracture clinic appointment? □ Yes (please continue to Q9b) □ No (please continue to Q10)
Q9b	What were these? □ Difficult to arrange or access transport to attend appointment □ Difficult to get time off e.g.: work or school/college to attend appointment □ Difficult to find someone to care for and look after a vulnerable person that I normally look after □ Difficult to attend appointment due to current medical condition or illness(es) □ Other, please specify: ....................................................................................................................................................
Q10	Did you feel you could understand the advice given during your fracture clinic appointment? □ Yes □ No
Q11	Did you feel you could follow the advice given during your fracture clinic appointment? □ Yes □ No
Q12	How satisfied did you feel about the outcome of your fracture clinic appointment? □ Very unsatisfied □ Unsatisfied □ Neutral □ Satisfied □ Very satisfied Why? ....................................................................................................................................................
Q13	Did you visit your GP or another hospital again with this Orthopaedic-related condition? □ Yes □ No

 Appendix 2

**Table 2:** Upper limb virtual fracture clinic guidelines. XROA = X-rays on arrival; w = week; OA = osteoArthritis; CMC = carpo-meta-carpal; STT = scapho-trapezio-trapezoid; MRI = magnetic resonance imaging; y = year; POP = plaster of Paris; MC = metacarpal; CMCJ = carpo-meta-carpal joint; CT = computed tomography; PIPJ = proximal interphalangeal joint; XR = X-rays; FDP = flexor digitorum profundus; DIPJ = distal interphalangeal joint

Diagnosis	Initial management	Follow-up
Clavicle undisplaced/Minimally displaced	Sling and analgesia	Discharge to physiotherapy
Clavicle displaced	Sling and analgesia	Upper limb clinic 4 w XROA
Clavicle distal third	Sling and analgesia	Upper limb clinic 2 w
Proximal humerus undisplaced/Minimally displaced	Sling and analgesia	Discharge to physiotherapy
Proximal humerus displaced	Sling and analgesia	Upper limb 2 w
Radial head Mason 1 & 2	Directly discharge to physiotherapy	Discharge
Radial head Mason 3	Discuss surgery	Upper limb 1–2 w
Scaphoid confirmed	Full scaphoid cast in nurse-led clinic	Follow up 4–6 w cast off XROA
Scaphoid suspected adult, no OA CMC/STT	Request urgent MRI/Futura splint or cast	See in clinic with report
Scaphoid suspected with OA CMC/STT	Splint	Discharge to hand therapy
Scaphoid suspected under 16 y	Review	2 w upper limb clinic
Distal radius fractures	Follow hospital protocol	Follow hospital protocol
Greenstick fracture (cortex discontinuity/plastic deformation)	POP 4w	Follow-up as required – can be nurse-led/doctor clinic
Torus (buckle) fracture - stable	Paediatric splint – nurse-led	Discharge
Dorsum triquetrum fracture	Futura splint or cast – nurse-led	Discharge or nurse-led for POP off 4 w
MC-5 Head	Consider surgery	Upper limb clinic
MC-5 Neck <70°	An ulnar gutter splint or Futura with neighbour strapping – nurse-led	Discharge or nurse-led 4 w
MC-5 shaft >30° or multiple fractures	Consider surgery	Upper limb
MC5 base extra-articular	Ulnar gutter/Futura splint – nurse-led	Discharge or nurse-led 4 w
MC5 base - CMCJ subluxation	Obtain joint-specific views or CT	Upper limb clinic
Phalangeal fractures	Follow hand clinic guidelines	Follow hand clinic guidelines
PIPJ dorsal dislocation/volar plate injury	Directly discharge to hand therapy	Hand therapy
PIPJ volar dislocation	Needs surgery	Hand clinic
Terminal phalanx fracture extra-articular	Mallet splint – nurse-led	Discharge
Terminal phalanx fracture with wound	Splint/Dressing – nurse-led	Nurse-led/Discharge
Terminal phalanx fracture intra-articular	See with XR in splint (FDP avulsion/DIPJ subluxation)	Upper limb
Bony Mallet	See with XR in splint (subluxation/non-union)	Upper limb
Soft tissue Mallet	Refer to hand therapy	Hand therapy
Boutoniere/Swan neck deformity	Hand clinic	Hand clinic
